# Biological Predictors of *De Novo* Tumors in Solid Organ Transplanted Patients During Oncological Surveillance: Potential Role of Circulating *TERT* mRNA

**DOI:** 10.3389/fonc.2021.772348

**Published:** 2021-10-21

**Authors:** Michela Cangemi, Stefania Zanussi, Enrica Rampazzo, Ettore Bidoli, Silvia Giunco, Rosamaria Tedeschi, Chiara Pratesi, Debora Martorelli, Mariateresa Casarotto, Ferdinando Martellotta, Ornella Schioppa, Diego Serraino, Agostino Steffan, Anita De Rossi, Riccardo Dolcetti, Emanuela Vaccher

**Affiliations:** ^1^ Department of Biomedical Sciences, University of Sassari, Sassari, Italy; ^2^ Immunopathology and Cancer Biomarkers, CRO Aviano, National Cancer Institute, IRCCS, Aviano, Italy; ^3^ Department of Surgery, Oncology and Gastroenterology, Section of Oncology and Immunology, University of Padova, Padova, Italy; ^4^ Cancer Epidemiology Unit, CRO Aviano, National Cancer Institute, IRCCS, Aviano, Italy; ^5^ Immunology and Diagnostic Molecular Oncology Unit, Veneto Institute of Oncology (IOV) – IRCCS, Padova, Italy; ^6^ Microbiology and Virology Unit, “S. Maria degli Angeli” Hospital, Pordenone, Italy; ^7^ Clinical Pathology, “S. Maria degli Angeli” Hospital, Pordenone, Italy; ^8^ Division of Medical Oncology A, Centro di Riferimento Oncologico (CRO) Aviano, National Cancer Institute, Istituto di Ricovero e Cura a Carattere Scientifico (IRCCS), Aviano, Italy; ^9^ Centre for Cancer Immunotherapy, Peter MacCallum Cancer Centre, Melbourne, VIC, Australia; ^10^ Sir Peter MacCallum Department of Oncology, The University of Melbourne, Melbourne, VIC, Australia; ^11^ Department of Microbiology and Immunology, The University of Melbourne, Melbourne, VIC, Australia; ^12^ Faculty of Medicine, The University of Queensland Diamantina Institute, Brisbane, QLD, Australia

**Keywords:** transplant, immunosuppression, oncological surveillance, cancer, circulating *TERT* mRNA, T cells

## Abstract

**Background:**

*De novo* tumors are a major cause of morbidity and mortality after long-term solid organ transplantation. Chronic immunosuppression strongly affects solid organ transplanted (SOT) patients’ immune system by promoting immune evasion strategies and reactivations of viruses with oncogenic potential, ultimately leading to cancer onset. In this scenario, an oncological Surveillance Protocol integrated with biobanking of peripheral blood samples and evaluation of immunovirological and molecular parameters was activated for SOT patients at CRO-IRCCS Aviano, with the aim of identifying suitable biomarkers of cancer development.

**Methods:**

An exploratory longitudinal study was designed based on two serial peripheral blood samples collected at least three months apart. Forty nine SOT patients were selected and stratified by tumor onset during follow-up. Spontaneous T-cell responses to EBV, CMV and tumor associated antigens, EBV-DNA and CMV-DNA loads, and circulating *TERT* mRNA levels were investigated.

**Results:**

Significantly higher levels of circulating *TERT* mRNA were observed 3.5-23.5 months before and close to the diagnosis of cancer as compared to tumor-free patients. Plasmatic *TERT* mRNA levels >97.73 copies/mL at baseline were significantly associated with the risk of developing *de novo* tumors (HR=4.0, 95%C.I. = 1.4-11.5, p=0.01). In particular, the risk significantly increased by 4% with every ten-unit increment in *TERT* mRNA (HR=1.04, 95%C.I. = 1.01-1.07, p=0.01).

**Conclusions:**

Although obtained in an exploratory study, our data support the importance of identifying early biomarkers of tumor onset in SOT patients useful to modulate the pace of surveillance visits.

## Introduction

Solid organ transplantation is currently recognized as the treatment of choice for patients with end-stage disease and the availability of potent anti-rejection drugs significantly reduced the occurrence of acute and chronic allograft rejections, even though long-term survival is still poor (1). Indeed, tumor development, viral infections/reactivations and cardiovascular complications are among the major causes of morbidity and mortality in solid organ transplanted (SOT) patients (2–4).

Combined with lifestyle habits, aging and concomitant comorbidities, chronic exposure to immunosuppressants plays a central role in the pathogenesis of these complications. The most common immunosuppressive drugs used after transplantation, calcineurin inhibitors (CNIs) and mTOR inhibitors (mTORi), while limiting the risk of allograft rejection, may have detrimental effects on antiviral and anti-tumor immunosurveillance. Indeed, CNIs, such as *Cyclosporine A* and *Tacrolimus*, exert their immunosuppressive action through the inhibition of the Calcineurin-NFAT signaling pathway, resulting in IL-2, TNFα, INF-γ downregulation and inhibition of T-cell activation and proliferation in response to foreign antigens (5–8). *Everolimus* and *Sirolimus* inhibit mTOR, a serine-threonine kinase involved in cell growth, proliferation, protein synthesis and apoptosis (9–11); they exert both immunosuppressant and anticancer activities. In particular, mTORi prevent dendritic cells maturation into antigen presenting cells, resulting in T-cell anergy and in the expansion of regulatory T-cells (12, 13).

In SOT patients under chronic immunosuppressive treatments, viral latent Epstein Barr virus (EBV) and/or Cytomegalovirus (CMV) reactivations can occur at any time after transplantation. In particular, CMV disease is the major cause of morbidity in this setting (14, 15). Chronic CMV infection is associated with functional alterations of the innate and adaptive arms of the immune system (16) with the expansion of terminally differentiated lymphocytes with reduced alloreactivity more evident with increasing age (17, 18). Hence, CMV reactivation and age potentially enhance pre-existing immunosuppression promoting immune escape in SOT patients. Moreover, the finding of CMV DNA and antigens in tumor cells from different types of cancer, such as colorectal cancer, malignant glioblastoma, EBV-negative Hodgkin lymphoma, prostatic carcinoma, and breast cancer, suggested an oncomodulatory role for this virus (19–22). EBV is involved in the pathogenesis of lymphoproliferative disorders and some epithelial tumors characterized by distinctive epidemiologic features and risk factors (23). Host immunity plays a crucial role in controlling EBV infection although the virus has evolved an elegant strategy to exploit B-cell differentiation and finally establish an asymptomatic latency in resting memory B lymphocytes (24). The iatrogenic impairment of host immunity against EBV may increase the risk to develop EBV-associated lymphoproliferative disorders, a heterogeneous group of diseases that may be a life-threatening complication after organ transplantation (25, 26).

The increased risk of developing tumors in SOT patients requires the activation of careful clinical and integrated laboratory follow-up protocols to detect cancer onset as early as possible. These strategies would greatly benefit from the availability of biomarkers that can reliably identify patients at high risk of developing tumors to be included in closer follow-up protocols. Monitoring EBV-DNA load coupled with the analysis of EBV-specific T-cell responses may be useful to identify patients at increased risk of EBV-driven lymphoproliferative disorders, while offering an indication for preemptive intervention (25). Under immunosuppressive conditions, latent CMV infection can reactivate and promote inflammatory responses that may contribute to cancer development (16–19). Nevertheless, the possible association between CMV reactivation and tumor onset in SOT recipients has been poorly investigated so far. Other candidate biomarkers have been identified that may be potentially useful to detect malignances early in SOT recipients. A polygenic risk score was recently associated with higher risk of non-melanoma skin cancers in patients receiving different solid organ transplants (27). The analysis of genome-wide DNA methylation of circulating T cells in kidney transplant recipients disclosed that a higher DNA methylation of SerpinB9, an intracellular inhibitor of granzyme B, was associated with the development of squamous cell carcinoma (28). Moreover, a significant reduction in Interleukin-27 expression and secretion by circulating immune cells was correlated with the risk of developing a malignancy in SOT recipients (29). Despite several efforts, however, the identification of reliable and clinically applicable biomarkers predictive of cancer risk in SOT recipients remains challenging due to the heterogeneity of cancers arising in this population and need of prospective series.

T-cell responses to tumor-associated antigens, particularly those specific for the so-called universal tumor associated antigens (TAAs) survivin and telomerase, may be detected in the blood of patients with various types of cancer, even in early phases of the disease (30–33). However, no information is currently available on the frequency and extent of T-cell responses to universal TAAs in SOT patients, either at the time of tumor diagnosis or at earlier time points.

Besides providing epitopes for the detection of specific T-cell responses, telomerase may also be regarded as an attractive molecular biomarker. In fact, more than 90% of all cancers acquire the capability to replicate indefinitely through the re-activation of telomerase, a ribonucleoprotein complex containing an internal RNA template and the catalytic protein telomerase reverse transcriptase (TERT), with telomere specific reverse transcriptase activity (34). *TERT* is the major rate-limiting catalytic subunit, which has a low/absent expression in normal cells but considerably high expression in the vast majority of tumor cells, suggesting that *TERT* expression level could be a specific biomarker for tumor development (25).

Here we report the results of a prospective exploratory study on advanced immunovirological and molecular monitoring carried out in a pilot cohort of SOT patients enrolled in a long term institutional cancer prevention program. With the main goal of identifying immunologic and/or virologic biomarkers potentially predictive of tumor development, serial blood samples were collected and investigated for EBV and CMV viremia, and the presence of T cell-responses specific for EBV and CMV viral epitopes and for universal TAAs. In addition, stimulated by the recently reported predictive and prognostic relevance of blood *TERT* mRNA levels in various clinical settings (35), we also investigated the circulating *TERT* mRNA levels as early marker of tumor development in SOT recipients.

## Materials and Methods

### Surveillance Protocol

Surveillance Protocol for SOT patients activated at the Centro di Riferimento Oncologico (CRO) in Aviano (PN), Italy, exploits a monitoring program focused on the most frequent and diagnosable *de novo* tumors with standardized screening (skin, lung, kidney, colorectum, cervix and pharynx carcinomas), and an integrated clinical follow-up. Moreover, the Surveillance Protocol includes a sub-protocol for translational research consisting in the biobanking of peripheral blood samples and in the evaluation of immunovirological and molecular parameters to identify candidate biomarkers predictive of *de novo* tumor development in SOT patients. The Surveillance Protocol was approved by the CRO Ethical Committee (ID number: CRO-2016-35). All study participants provided informed written consent at the enrolment. European and National ethical guidelines for research involving human subjects were respected. Criteria of inclusion in the Surveillance Protocol were: to have received a solid organ transplantation at least one year before the enrollment, age ≥18 years old, ECOG 0-2 performance status, life expectancy ≥6 months, and regular follow-up compliance. Subjects with pre-transplant tumors different from non-melanoma skin cancer, Tis cervix, and hepatocellular carcinoma (HCC) in liver transplant recipients were excluded, as well as subjects with complete remission <3 years or post-transplant and pre-enrolment active tumors. Moreover, patients have been considered not eligible for the study if showing the following severe co-morbidities at enrolment or in the previous year: heart failure, myocardial infarction, stroke, severe hepatic and/or renal failure, tuberculosis, psychiatric pathology. The appearance of these clinical conditions during surveillance was also considered as reason for withdrawal from the program along with organ rejection or return to dialysis, the development of advanced tumors requiring chemo and/or radiotherapy or treatment with major root surgery, and the occurrence of life-threatening chronic infections.

The pace of surveillance was established grounding on the classification of the patients by tumor risk. Patients were assigned to the high-risk group if they showed at least one of the following characteristics: duration of immunosuppression ≥10 years, age at transplant ≥50 years, metachronous transplants (i.e., multiple non-synchronous transplants), abuse of smoking/tobacco/alcohol within 15 years from enrolment in the surveillance program, presence of HIV infection. High-risk patients followed an intensive clinical surveillance focused on the diagnosis, by standardized screening protocols, of the more frequent *de novo* tumors, such as carcinoma of the skin, lung, kidney, liver, colorectal, cervix, and head-neck/esophagus. Low-risk patients followed the general population guidelines. Breast and prostate cancer screening complied the general population guidelines in both high and low-risk groups. Unless the patient did not access the visit for personal or health reasons, the clinical assessment was performed every six months for high-risk and annually for low-risk patients. Peripheral blood samples for the immunovirological and molecular surveillance were collected at each visit and close to the date of histological examination that defined the cancer diagnosis.

### Sample Collection

Peripheral blood samples were processed within four hours from blood withdrawal. Two aliquots of fresh EDTA peripheral whole blood were immediately stored at -80°C. Thereafter, blood was centrifuged at 800 rpm for 10 minutes and the plasma fraction was further centrifuged at 2100 rpm for 15 minutes, aliquoted in two vials and frozen at -80°C. Peripheral blood mononuclear cells (PBMCs) to be used in functional assays were isolated by Ficoll-Hypaque gradient centrifugation, washed once in PBS, counted by ADAM Cell Counter (DigitalBio), resuspended in 1 mL of FCS containing 10% DMSO and, finally, stored at -120°C.

### Biological Study Design

Among 109 SOT patients under surveillance from 2015 to 2018, 49 were selected for an exploratory longitudinal research design based on the availability of two serial peripheral blood samples collected for laboratory analyses at least three months apart and no evidence of tumor onset between enrolment in the Surveillance Protocol and the first sampling. The first and the second blood withdrawal will henceforth be referred to as baseline and follow-up, respectively. Patients’ characteristics of this sub-cohort at the time of enrolment in the Surveillance Protocol were described in **Supplementary Table 1**. The biological parameters studied were: antigen-specific T-cell responses against two “universal” TAAs-derived peptide mixes (Survivin and TERT) and viral peptide pools (EBV, CMV), and whole blood CMV and EBV viremia. Quantification of plasma *TERT* mRNA was also evaluated.

### ELISpot Assay

Virus and tumor antigen-specific T cell responses were investigated by using an interferon (IFN)-γ enzyme-linked immunosorbent spot (ELISpot) commercial assay (“Human IFN-γ Single Color ELISPOT”, ImmunoSpot^®^, Cellular Technology Limited (CTL), OH, USA), according to the manufacturer’s instructions. Briefly, ninety-six-well plates were pre-coated by an overnight incubation at 4°C with 2μg/mL anti-human IFN-γ capture antibody. The next day, PBMCs were thawed and washed once in serum free RPMI 1640, counted and resuspended in CTL-test Medium at a concentration of 10.5x10^6/mL cells. CMV, EBV, Survivin, TERT peptide mixes (ProImmune, Oxford UK; 0.2ng/mL of each peptide mix) or unspecific stimuli (0.5mg/mL αCD3/αCD28) were resuspended in CTL-test Medium, plated in triplicate and incubated for 10-20 minutes. Triplicate wells without stimulus were used as negative control. Next, patient’s PBMCs were placed in co-culture at a concentration of 500,000 PBMCs/well and incubated overnight. The next day, spots were detected with anti-human IFN-γ (biotin) streptavidin alkaline phosphatase, and Blue Developer Solution. Spots were counted and analyzed by using the Immunospot^®^ plate scanning and analysis service (CTL-Europe GmbH, Bonn, Germany).

### EBV and CMV Viral Load

For EBV viral load evaluation, cryo-preserved aliquots of 200 μl whole blood were processed for DNA extraction with the QIAamp Blood Mini kit (Qiagen, Gmbh, Hilden, Germany) within 15 days from collection, following the instructions from the manufacturer. A final elution volume of 50 μL was used and EBV-DNA was quantified by real time TaqMan PCR by using the ABI PRISM 7900 HT Sequence Detection System (Applied Biosystems), as previously described (36, 37). EBV viral load was expressed as copies of EBV-DNA genomes per milliliter of whole blood. For statistical analyses, a viral load of zero copies/mL was assigned to samples with undetectable EBV-DNA.

CMV viral load was assessed by the Abbott RealTime CMV assay and the automated m2000 RealTime system (Abbott Molecular Inc., IL, USA) according to manufacturer’s instructions. For statistical analyses, a CMV viral load of 39 copies/mL was assigned to samples with detectable CMV-DNA, but below the threshold (40 copies/mL); a viral load of zero copies/mL was assigned to samples with undetectable CMV-DNA.

### Quantification of Circulating *TERT* mRNA

RNA was extracted from plasma samples as previously described (38, 39), using 1 mL instead of 500 μL of plasma and reagents’ quantities adjusted accordingly. RNA was reverse transcribed into cDNA using the SuperScript TM III RNase reverse transcriptase assay (Thermo Fisher Scientific) in a final volume of 80 μL, according to the manufacturer’s instructions.

The expression of *TERT* transcripts in the plasma samples was quantified by real-time PCR, as previously described (38). Briefly, the primers AT1 (5′-CGGAAGAGTGTCTGGAGCAA-3′) and AT2b (5′-CGCAGCTGCACCCTCTTCA-3′), which bind to nucleotide sequences located upstream of the RT motif 1 on the *TERT* gene, thus allowing amplification of all *TERT* transcripts, and the fluorogenic probe AT (FAM 5′-TTGCAAAGCATTGGAATCAGACAGCAC-3′ TAMRA) recognizing the sequence located inside the product amplified by AT1/AT2b were employed (38). The PCR was performed using an ABI prism 7900 Sequence Detection System (PE Applied Biosystems, Foster City, CA, USA) in 50 μL of mixture containing 25 μL 2x TaqMan universal master mix (PE Applied Biosystems), 100 nM of fluorogenic probe, 600 nM of primer AT1, 900 nM of primer AT2b and 10 μl of cDNA sample. After 2 min at 50°C, to allow the uracil *N*-glycosylase to act, and a denaturation step lasting 10 min at 95°C, 50 cycles were run, each consisting of 30 s at 95°C, 30 s at 60°C and 30 s at 72°C. Each sample was run in triplicate and the mean Ct values were plotted against the standard *TERT* reference curve, which was generated with serial fivefold dilutions of the *TERT* amplicon, as previously described (40). *TERT* values were estimated per mL according to the X8 conversion factor and then expressed as *TERT* copies per mL.

### Statistical Analysis

Comparisons of unmatched, and baseline-follow-up matched continuous variables were made using the non-parametric Mann-Whitney U test and the Wilcoxon signed rank test, respectively. Fisher’s exact test was computed for discrete variables when appropriate. Successively, the impact of biological factors on tumor onset probability was assessed. Due to the exploratory nature of this study, Receiver Operating Characteristic (ROC) curves were calculated for continuous clinical and biological covariates at baseline to determine the best cut-off value that differentiated the risk of tumor onset with the highest specificity and sensitivity (41) (**Supplementary Table 2**). Time-to-tumor-onset was calculated from the date of baseline to the date of tumor diagnosis. Subjects who did not develop any tumor were censored at the date of follow-up. Time of immunosuppression was computed from the time of the first transplant to the date of baseline. Tumor onset probability was examined by means of the Kaplan-Meier method (42), and risk was quantified by means of univariate and multivariate Cox proportional hazard models. Hazard Ratio (HR) and corresponding 95%C.I.s were calculated by dichotomizing continuous clinical and biological variables by the cut-off assessed through the ROC curve. Age was categorized by the median value of the overall cohort and included for HR adjustment. Moreover, HR was computed for ten-unit increases in the level of *TERT* mRNA. Analyses were performed by means of SAS, version 9.4 (SAS Institute Inc., Cary, NC, 2002–2008). All statistical tests were considered statistically significant at a two-sided *p*-value <0.05.

## Results

### Patients Characteristics

**Table 1** shows the main patients’ demographic and clinical characteristics of the 49 SOT patients at baseline: median age was 60 (31–80) years, 32 (65.3%) were males and 17 (34.7%) were females; forty subjects (81.6%) were kidney transplanted and nine (18.4%) heart, liver or heart plus kidney transplanted. Thirty-eight (77.6%) were treated with CNI, four (8.2%) with mTORi and seven with CNI plus mTORi (14.3%). The median immunosuppression duration from first transplantation was 10.5 (1.2-28.4) years, while six patients received adjunctive pre-transplant immunosuppressive therapy.

**Table 1:** Baseline demographic and clinical characteristics of the 49 SOT patients.

	Total N = 49	NT N = 33	T N = 16
**Age**	60 (31-80)	58 (31-79)	
Median years (range)			64 (46-80)
p-value°			0.05
**Gender**			
M, n (%)	32 (65.3)	20 (60.6)	12 (76.5)
F, n (%)	17 (34.7)	13 (39.4)	4 (23.5)
p-value*			0.36
**Transplanted Organ**			
Kidney, n (%)	40 (81.6)	26 (78.8)	14 (87.5)
Liver, heart or heart+kidney, n (%)	9 (18.4)	7 (21.2)	2 (12.5)
p-value*			0.70
**Type of Immunosuppressive therapy**			
CNI, n (%)	38 (77.6)	26 (78.8)	12 (75.0)
mTOR/mTOR + CNI, n (%)	11 (22.4)	7 (21.2)	4 (25.0)
p-value*			1.00
**Time of Immunosuppression**	10.5 (1.2-28.4)	10.2 (1.2-24.6)	11.8 (3.1-28.4) 0.36
Median years (range)			
p-value°			
**Pre-transplant immunosuppressive therapy**			
No (%)	43 (87.8)	29 (87.9)	14 (87.5)
Yes (%)	6 (12.2)	4 (12.1)	2 (12.5)
p-value*			1.00

NT, no tumor cohort; T, tumor cohort; M, males; F, females; °, Mann-Whitney U-test; *, Fisher exact test.

During surveillance and after a median time of 10.4 (3.5-23.6) months from baseline, 16 patients were included in the tumor cohort (T) as the following *de novo* tumors were diagnosed: 13 BCC or SCC, one melanoma *in situ*, one indolent non-Hodgkin lymphoma and one renal carcinoma (**Supplementary Table 1**). The median time between baseline and follow-up sampling was 11.7 (6.0-24.2) months for patients who developed a tumor and 12.2 (5.8-28.8) months for those tumor-free (non-tumor cohort, NT) (p=0.13). Cancer was diagnosed before or after a maximum of 3.7 months from the second sample, which was therefore indicative of an underlying neoplastic condition. Patients of the T cohort were significantly older than subjects of the NT cohort (p=0.05). No statistically significant difference was observed in the distribution of the SOT patients for the other parameters analysed.

### Immunovirological and Molecular Analyses

**Figure 1:**
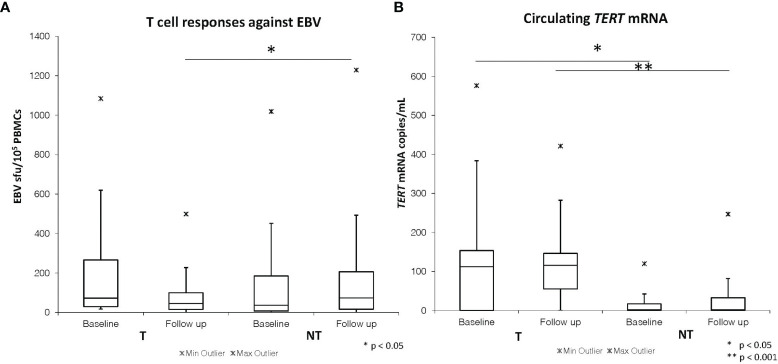
Baseline and follow-up EBV ELISpot T cell responses **(A)** and plasma *TERT* mRNA levels **(B)** in SOT patients developing (T) and not developing tumors (NT).

**Table 2** summarizes the baseline and follow-up median values of the immunovirological and molecular parameters assessed in the SOT patients after stratification by tumor occurrence. No statistically significant difference was found in EBV- and CMV-specific T-cell responses between baseline and follow-up samples within both the NT and the T cohort. Patients in the T cohort showed significantly decreased levels of EBV-specific circulating T cells in the samples collected close to cancer diagnosis when compared with the follow-up samples from the NT cohort [median (range) T *vs.* NT: 45 (0–499) *vs.* 144 (1-1229) sfu/10^5 PBMCs, p=0.02] (**Figure 1A** and **Table 2**).

**Table 2:** Biological parameters in the 49 SOT patients after stratification by tumor occurrence.

	NT N = 33		T N = 16	
	*Baseline*	*Follow-up*	*Baseline*	*Follow-up*
**T cell responses against EBV**				
Median sfu/10^5 PBMCs (range)	139 (2-1019)	144 (1-1229)	72 (17-1084)	45 (0-499)
p-value (Mann-Whitney test)°			0.53	0.016
p-value (Wilcoxon test)^‡^		0.30		0.35
**T cell responses against CMV**				
Median sfu/10^5 PBMCs (range)	539 (1-5000)	614 (1-5000)	521 (13-1097)	501 (0-1726)
p-value (Mann-Whitney test)°			0.53	0.32
p-value (Wilcoxon test)^‡^		0.73		0.87
**EBV-DNA**				
Undetected, n (%)	18 (54.6)	18 (54.6)	6 (37.5)	6 (37.5)
Detected, n (%)	15 (45.4)	15 (45.4)	10 (62.5)	10 (62.5)
p-value (Fisher exact test)*			0.36	0.36
Median copies/mL (range)	0 (0-8845)	0 (0-4334)	86 (0-3135)	38 (0-3485)
p-value (Mann-Whitney test)°			0.38	0.80
p-value (Wilcoxon test)^‡^		0.44		0.38
**CMV-DNA^$^**				
Undetected, n (%)	27 (87.1)	28 (84.9)	12 (75.0)	13 (81.3)
Detected, n (%)	4 (12.9)	5 (15.1)	4 (25.0)	3 (18.7)
p-value (Fisher exact test)*			0.42	0.71
Median copies/mL (range)	0 (0-655)	0 (0-670)	0 (0-81)	0 (0-79)
p-value (Mann-Whitney test)°			0.54	0.89
p-value (Wilcoxon test)^‡^		N.E.		N.E.
**T cell responses against SURVIVIN^$^**				
Median sfu/10^5 PBMCs (range)	9 (0-1329)	14 (0-1209)	14 (1-538)	10 (0-987)
p-value (Mann-Whitney test)°			0.73	0.53
p-value (Wilcoxon test)^‡^		0.08		0.83
**T cell responses against TERT^$^**				
Median sfu/10^5 PBMCs (range)	9 (0-1216)	10 (0-971)	11 (1-1196)	12 (0-378)
p-value (Mann-Whitney test)°			0.53	0.91
p-value (Wilcoxon test)^‡^		0.13		0.94
***TERT* mRNA**				
Median copies/mL (range)	0 (0-120)	0 (0-206)	112 (0-576)	115 (0-421)
p-value (Mann-Whitney test)°			0.03	<0.001
p-value (Wilcoxon test)^‡^		0.90		0.60

NT, no tumor cohort; T, tumor cohort; °, Mann-Whitney U test (no tumor vs. tumor cohort); ‡, Wilcoxon paired signed-rank test (baseline vs. follow-up values); *, Fisher exact test (no tumor vs. tumor cohort); N.E., not evaluable; $, the sum does not add up to the total because of missing values; sfu/10^5 PBMCs, spot forming units/10^5 Peripheral Blood Cells.

The percentage of SOT patients with detectable EBV-DNA did not change at baseline compared to follow-up within both the T and NT cohorts, with no statistically significant difference in viral load values throughout the time. No statistically significant difference was found between T and NT cohorts neither for EBV-DNA positivity rate nor for EBV-DNA levels neither at baseline [T *vs.* NT: 62.5% *vs.* 45.4%, p=0.36; median (range) 86 (0-3135) copies/mL *vs.* 0 (0-8845) copies/mL, p=0.38] nor at follow-up [T *vs.* NT: 62.5% *vs.* 45.4%, p=0.36; median (range) 38 (0-3485) copies/mL *vs.* 0 (0-4334) copies/mL, p=0.80].

The percentage of SOT patients with detectable CMV viremia and the CMV-DNA levels did not change from baseline to follow-up in the T and NT cohorts. There was no statistically significant difference in CMV-DNA positivity rate and CMV-DNA load between the T and NT cohorts at baseline [25.0% *vs.* 12.9%, p=0.42; median (range) 0 (0-81) *vs.* 0 (0-655), p=0.54] and at follow-up time [18.7% *vs.* 15.1%, p=0.71; median (range) 0 (0-79) *vs.* 0 (0-670), p=0.89].

The levels of Survivin and TERT-specific T-cells were similar in the NT and T cohorts at baseline [median (range) TAA reactivity in T *vs.* NT: 14 (1-538) *vs.* 9 (0-1329), p=0.73 for Survivin; 11 (1-1196) *vs.* 9 (0-1216), p=0.53 for TERT] or follow-up [median (range) TAA reactivity in T *vs.* NT: 10 (0-987) *vs.* 14 (0-1209), p= 0.53 for Survivin; 12 (0-378) *vs.* 10 (0-971), p=0.91 for TERT]. No significant changes in TAA-specific circulating T cell levels were observed over time (from baseline to follow-up) within each group.

Both T and NT cohorts of SOT patients showed no statistically significant differences in circulating cell-free *TERT* mRNA levels when comparing baseline to follow-up time. However, significantly higher levels of circulating *TERT* mRNA were detected at baseline in patients belonging to the T cohort [112 (0-576) copies/mL] as compared to those from the NT cohort [0 (0-120) copies/mL, p=0.03] (**Table 2** and **Figure 1B**). These findings suggest that a significant increase in the levels of plasmatic *TERT* mRNA can be detected in transplanted patients several months (range 3.5-23.5 months) before the diagnosis of cancer. Moreover, patients in the T cohort showed significantly higher levels of circulating *TERT* mRNA also in the samples obtained close to the date of cancer diagnosis [T *vs.* NT cohort: 115 (0-421) copies/mL *vs.* 0 (0-206) copies/mL, p<0.001] (**Table 2** and **Figure 1B**).

### Potential Clinical and Biological Predictors of Tumor Occurrence

**Figure 2:**
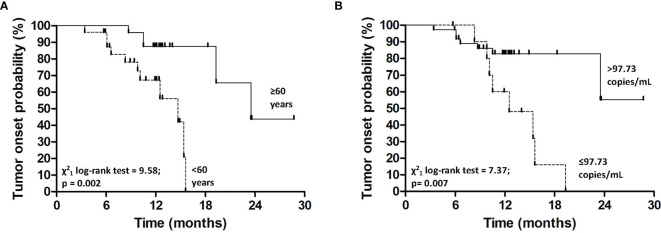
Kaplan-Meier estimates for tumor onset probability according to age **(A)** and plasma *TERT* mRNA levels at baseline **(B)**.

We evaluated the potential demographic, clinical and biological predictors of tumor occurrence for SOT patients at baseline. We found that patients ≥60 years had a higher likelihood to develop tumors as compared to patients <60 years (Log-Rank test=9.58; p<0.01) (**Figure 2A**). More specifically, patients ≥60 years presented a higher risk of tumor onset than younger (HR=6.7 for patients ≥60 *vs.* <60 years, 95%C.I. = 1.7-22.6, p<0.01) (**Table 3**).

**Table 3:** Cox regression analysis evaluating the associations between baseline demographic, clinical or biological parameters and tumor onset.

	NT N = 33 n. (%)	T N = 16 n. (%)	HR (95%C.I.)	p-value	HR* (95%C.I.)	p-value
**Age, years**						
<60	14 (42.4)	11 (68.8)	1^ƚ^		–	
≥60	19 (57.6)	5 (31.3)	6.7 (1.7-22.6)	<0.01	–	–
**Gender**						
M	20 (60.6)	12 (76.5)	1^ƚ^		1^ƚ^	
F	13 (39.4)	4 (23.5)	0.7 (0.2-2.2)	0.52	0.6 (0.2-1.8)	0.31
**Transplanted organ**						
Kidney	26 (78.8)	14 (87.5)	1^ƚ^		1^ƚ^	
Liver/heart/heart + kidney	7 (21.2)	2 (12.5)	0.9 (0.2-4.2)	0.93	0.4 (0.1-2.1)	0.31
**Type of immunosuppressive therapy**						
CNI	26 (78.8)	12 (75.0)	1^ƚ^		1^ƚ^	
mTOR/mTOR + CNI	7 (21.2)	4 (25.0)	1.3 (0.4-4.2)	0.62	1.2 (0.4-3.7)	0.82
**Time of Immunosuppression, years**						
≤18.83	31 (93.9)	11 (68.8)	1^ƚ^		1^ƚ^	
>18.83	2 (6.1)	5 (31.3)	2.4 (0.8-7.1)	0.10	2.1 (0.7-6.2)	0.18
**Pre-transplant immunosuppressive therapy**						
No	29 (87.9)	14 (87.5)	1^ƚ^		1^ƚ^	
Yes	4 (12.1)	2 (12.5)	2.0 (0.4-9.4)	0.37	2.0 (0.4-9.4)	0.38
**EBV-DNA, copies/mL**						
≤29	19 (57.6)	6 (37.5)	1^ƚ^		1^ƚ^	
>29	14 (42.4)	10 (62.5)	2.6 (0.9-7.4)	0.07	2.0 (0.7-5.9)	0.19
**CMV-DNA^$^, copies/mL**						
Undetected	27 (87.1)	12 (75.0)	1^ƚ^		1^ƚ^	
Detected	4 (12.9)	4 (25.0)	1.6 (0.5-4.9)	0.45	1.7 (0.5-5.5)	0.36
**T cell responses against EBV^$^, sfu/10^5 PBMCs**						
>106	13 (39.4)	9 (64.3)	1^ƚ^		1^ƚ^	
≤106	20 (60.6)	5 (35.7)	2.3 (0.8-6.9)	0.14	2.0 (0.7-6.1)	0.23
**T cell responses against CMV^$^, sfu/10^5 PBMCs**						
>1097	7 (21.2)	1 (7.1)	1^ƚ^		1^ƚ^	
≤1097	26 (78.8)	13 (92.9)	0.6 (0.7-46.1)	0.10	3.9 (0.4-34.8)	0.22
***TERT* mRNA^$^, copies/mL**						
≤97.73	30 (90.9)	7 (46.7)	1^ƚ^		1^ƚ^	
>97.73	3 (9.1)	8 (53.3)	4.0 (1.4-11.5)	0.01	2.5 (0.8-7.8)	0.13
10-unit increases			1.04 (1.01-1.07)	0.01	1.02 (0.99-1.06)	0.22
**T cell responses against TERT^$^, sfu/10^5 PBMCs**						
≤8	16 (48.5)	4 (28.6)	1^ƚ^		1^ƚ^	
>8	17 (51.5)	10 (71.4)	1.4 (0.4-4.7)	0.58	1.7 (0.5-5.8)	0.37
**T cell responses against SURVIVIN^$^, sfu/10^5 PBMCs**						
≤13	20 (60.6)	7 (50.0)	1^ƚ^		1^ƚ^	
>13	13 (60.6)	7 (50.0)	1.2 (0.4-3.4)	0.78	1.4 (0.5-4.1)	0.59

NT, no tumor cohort; T, tumor cohort; HR, Hazard Ratio; C.I., Confidence Interval; *, Adjusted for age; ƚ, reference category; $, the sum does not add up to the total because of missing values; sfu/10^5 PBMCs, spot forming units/10^5 Peripheral Blood Cells.

Kaplan-Meier’s evaluation showed that patients with baseline circulating *TERT* mRNA levels above 97.73 copies/mL had a significant higher risk to develop tumors than patients with baseline *TERT* mRNA levels below this value (Log-Rank test=7.37; p<0.01) (**Figure 2B**). Accordingly, the risk of developing tumors was significantly higher in individuals with high baseline circulating *TERT* mRNA levels than patients with lower values (HR=4.0 for patients with >97.73 *vs.* ≤97.73 copies/mL, 95%C.I. = 1.4-11.5, p=0.01). The area under the ROC curve, sensibility, and specificity for this parameter were 0.70 (95% C.I. = 0.60-0.82), 0.53 (95%C.I. = 0.27-0.79), and 0.94 (95%C.I. = 0.80-0.99), respectively. Notably, every ten-unit increment of *TERT* mRNA was associated with a 4% increase in the risk of developing cancer (HR=1.04, 95%C.I. = 1.01-1.07, p=0.01) (**Table 3**). After adjustment for age, the association of *TERT* mRNA levels above the cut-off with the risk of tumor development was still high, but not significant (HR=2.5, 95%C.I. = 0.8-7.8, p=0.13). The risk of tumor development for patients over 60 years of age raised with increasing TERT mRNA levels [HR and 95%C.I. for patients over 60 years of age and *TERT* mRNA ≤97.73 or >97.73 copies/mL *vs.* patients under 60 and *TERT* mRNA ≤97.73 copies/mL=6.7 (1.1-40.4) or 12.3 (2.3-64.7)] (**Table 4**).

**Table 4:** HR and 95%C.I. according to the combined effect of age and circulating *TERT* mRNA levels among 49 SOT patients.

Age, years	*TERT* mRNA copies/mL					
	≤97.73			>97.73		
	N	HR	95% C.I.	N	HR	95% C.I.
<60	21	1^ƚ^	–	3	4.9	0.7-35.7
≥60	16	6.7	1.1-40.4	8	12.3	2.3-64.7

HR, Hazard Ratio; C.I., Confidence Interval; ƚ, reference category.

EBV-DNA higher than 29 copies/mL at baseline was associated to a higher, although not significant, risk to develop tumors (Log-rank test=3.51 for subjects with >29 *vs.* ≤29 copies/mL, p=0.06 (not shown); HR=2.6, 95%C.I. = 0.9-7.4, p=0.07, **Table 3**).

## Discussion

The identification of suitable biomarkers able to predict the risk of impending tumor development in SOT patients constitutes an important but still unmet clinical need. To address this relevant issue, we took advantage of the clinical and laboratory surveillance program for SOT patients recently activated at CRO-IRCCS Aviano. The routine clinical workup of these patients was implemented with the investigation of CD8 T-cell responses against EBV and CMV antigens and “universal” TAAs, the assessment of viral reactivations and quantification of circulating *TERT* mRNA in plasma as potential source of risk-predictive biomarkers for a broad spectrum of cancers, such as those occurring in SOT recipients. Here we report the results of the first prospective cohort of patients.

CMV and EBV infections are highly prevalent in the general population, and the immunosuppressive treatment of SOT recipients can occasionally trigger viral reactivations that directly or indirectly may enhance the risk of cancer development. In our series, CMV viremia was detected in a low fraction of cases (approximately 17%), consistent with a relatively infrequent CMV reactivation, which occurred at comparable frequency in patients of the T and NT cohorts. Similarly, the two groups of SOT recipients showed no significant difference in the extent of CMV-specific T cell responses, ruling out any possible pathogenic association between CMV reactivation and the occurrence of tumors. It should be considered, however, that the majority of tumors observed in our cohort were non-melanoma skin cancers, suggesting that these results warrant a confirmation in larger prospective series including higher numbers of non-skin tumors. By contrast, about half of the SOT recipients investigated had detectable EBV viremia, indicating a relatively more frequent reactivation of EBV. Comparative analysis of the T and NT cohorts did not disclose significant differences in the extent of EBV-specific T-cell responses, except for the significantly lower levels of circulating EBV-specific T-cells detected at the time of cancer diagnosis in the T cohort compared to the NT cohort samples at follow-up. This intriguing observation could be the result of additional immunosuppression imposed by cancer onset and/or the diversion of residual immune responses towards cancer-associated antigens different from Survivin or TERT and warrants further investigation in larger series. The fact that we did not observe significantly increased levels of T-cell responses to EBV is consistent with the observation that, in our series, no patient developed EBV-related lymphoproliferations, thus preventing the possibility to assess the predictive value of this analysis. Indeed, our results are in line with the observation that EBV-DNA load is generally high in the first year after transplantation in SOT patients with positive EBV-specific T cell responses, when the risk of EBV-driven lymphoproliferative disorders is high (43).

Despite T cell responses to “universal” TAAs can be detected also in patients with early stages of cancer (30, 31), no significantly higher levels of T-cells specific for TERT and Survivin were detected at baseline or at the time of diagnosis of cancer in the blood of T and NT patients. Globally, IFN-γ T cell responses against TERT and Survivin were not significantly different in T and NT cohorts also when values over time (i.e., baseline *vs.* follow-up) were considered. This could be due to the degree of variability of antigen-specific T cell responses among patients, as frequently observed in the cancer setting (44). The negative impact on tumor antigen priming potentially exerted by immunosuppressive drugs could also at least in part explain these findings, in particular considering that CNIs, the most frequently used drugs administered to our cohort of SOT patients, were shown to markedly inhibit antigen presentation through both MHC class I and II (45).

Expression of TERT, which is usually repressed in normal somatic cells, is essential to sustain the unlimited replicative potential of cancer cells (34) showing a critical role in tumor formation and progression. Consistently with this critical pathogenic role, circulating cell-free *TERT* mRNA can be detected in plasma from cancer patients at levels that significantly correlate with those in tumor specimens (46), conversely, cell-free *TERT* mRNA is not detectable in plasma samples of healthy volunteers (46, 47). Importantly, several studies have been demonstrated that circulating *TERT* mRNA is an independent prognostic marker in different types of tumors (35), including gastric (48), prostatic (49), lung (50), and colorectal cancers (38, 46, 51). In addition, *TERT* mRNA levels in plasma samples of patients with rectal cancer were identified as a predictive marker of response to therapy (38, 39, 51). In the present study, we found that patients of the T cohort showed significantly higher levels of circulating *TERT* mRNA than those of the NT cohort both at baseline and follow-up. These findings are consistent with the evidence that *TERT* expression is a hallmark of cancer (34). Our observation that the levels of circulating *TERT* mRNA were significantly higher even before the diagnosis of cancer in the T cohort is intriguing and strongly suggests the potential clinical relevance of the inclusion of circulating *TERT* mRNA among the biomarkers to be investigated for monitoring of SOT recipients. Indeed, the univariate analysis shows that the risk of developing tumors was significantly higher in SOT recipients with high baseline circulating *TERT* mRNA levels than those with low values. The risk of tumor development in these patients remained high after adjusting for age even not at significant level as a probable consequence of the relatively limited sample size.

Considering that the majority of tumors occurred in our series of SOT recipients included non-melanoma skin cancers, our results suggest that monitoring the circulating *TERT* mRNA levels could identify SOT patients requiring a more frequent clinical and dermatologic follow-up. The need of non-invasive biomarkers for the management of BCC and SCC in SOT recipients is remarkably important given the high incidence of these malignancies in the post-transplant setting (52–55). It is noteworthy that non-melanoma skin cancers in SOT recipients tend to be more aggressive, with higher morbidity and mortality compared to the general population (56–59). Moreover, careful monitoring of circulating *TERT* mRNA could be helpful in pre-transplantation to define the minimum non-melanoma skin cancer remission times before the graft, due to the high rate of post-transplant relapse in the patients with pre-transplant skin malignancies (60, 61).

Because of the exploratory nature of this report, all types of cancer developed during surveillance were described instead of focusing on non-melanoma skin cancers only. Further studies in independent prospective cohorts will be however necessary to clinically validate the possible role of circulating *TERT* mRNA levels as predictor of non-melanoma skin cancers. Moreover, analysis of larger cohorts of SOT recipients developing tumors different from non-melanoma skin cancers is warranted to establish whether circulating *TERT* mRNA levels can serve as a global early marker of tumor development in this setting. Finally, it should be kept in mind that, despite the fact that in most of the tumors replicative immortality is sustained by the inappropriate re-activation of TERT, a small percentage of neoplasms (approximately 10-15%), mainly those of mesenchymal and neuroepithelial origin, grow independently from TERT/telomerase. In these tumors, telomere shortening that accompanies cell proliferation is compensated by the alternative lengthening of telomeres (ALT) mechanism, a homologous recombination-based process (62, 63). For the ALT-dependent neoplasms occurring in SOT patients, circulating TERT mRNA detection would not be informative, therefore other blood-based biomarkers of tumor development need to be investigated.

Our results, even if preliminary and on a relatively small cohort, emphasize the relevance of the implementation of a specific program of oncological monitoring for SOT patients, which considers the different variables present in such complex patients. Monitoring programs should be integrated with various investigative strategies that can identify and prospectively validate markers predictive of *de novo* tumors, to be combined with already established approaches that help identify high-risk patients. To the best of our knowledge, this is the first comprehensive immunovirological and molecular monitoring study in a prospective cohort of SOT patients aimed at identifying such biomarkers. The results obtained in this pilot series, although not conclusive, are consistent with the hypothesis that the detection of early tumor markers, such as increased levels of circulating *TERT* mRNA, may be of help to assess the risk of cancer in SOT patients.

## Data Availability Statement

The raw data supporting the conclusions of this article will be made available by the authors, without undue reservation.

## Ethics Statement

The studies involving human participants were reviewed and approved by Ethical Committee of the Centro di Riferimento Oncologico, Aviano, Italy. The patients/participants provided their written informed consent to participate in this study.

## Author Contributions

MC participated in performing the experiments, data curation and analysis, drafting and editing the work, and final approval. SZ participated in performance of the research, data acquisition, curation, analysis and presentation, drafting, revising and editing the work, and final approval. ER participated in performing the experiments, revising the work, and final approval. EB Participated in data analysis and presentation, revising the work, and final approval. SG participated in performing the experiments, revising the work, and final approval. RT participated in performing the experiments, revising the work, and final approval. CP participated in performing the experiments, data analysis, revising the work, and final approval. DM participated in performing the experiments, revising the work, and final approval. MTC participated in data curation, revising the work, and final approval. FM participated in performance of the research, and final approval. OS participated in performance of the research, and final approval. DS participated in funding acquisition and final approval. AS participated in supervision, resources acquisition, and final approval. AR participated in conception of the work, funding and resources acquisition, drafting, revising and editing the work, supervision and final approval. RD participated in conception of the work, funding acquisition, drafting, revising and editing the work, supervision and final approval. EV participated in project administration, data acquisition and curation, revising and editing the work, supervision and final approval.

## Funding

This work was partly supported by grants from the Italian Ministry of Health (Ricerca Corrente), Associazione Italiana per la Ricerca sul Cancro (AIRC) [Grant no. IG-19112, PI DS], Department of Surgery, Oncology and Gastroenterology (DiSCOG), University of Padova, Italy [Grant no. BIRD181981/18, PI AR], Cancer Council Queensland [APP1145758, APP1165063, PI RD].

## Conflict of Interest

The authors declare that the research was conducted in the absence of any commercial or financial relationships that could be construed as a potential conflict of interest.

## Publisher’s Note

All claims expressed in this article are solely those of the authors and do not necessarily represent those of their affiliated organizations, or those of the publisher, the editors and the reviewers. Any product that may be evaluated in this article, or claim that may be made by its manufacturer, is not guaranteed or endorsed by the publisher.
