# Supplementary material for: Biological Predictors of De Novo Tumors in Solid Organ Transplanted Patients During Oncological Surveillance: Potential Role of Circulating TERT mRNA
Source: Front Oncol. 2021 Oct 21;11:772348. doi: 10.3389/fonc.2021.772348 (PMC8567137; doi:10.3389/fonc.2021.772348)
Supplement: Supplementary file 1 [file Table_1.docx]

Supplementary Table 1: Clinical characteristics of the 49 SOT patients at enrolment in the Surveillance Protocol, with indication of the subsequent tumor developed

| **Patient** | **Age** | **Sex** | **Transplantated organ** | **Years of IS** | **Type of IS** | **Risk group** | **Developed tumor** |
| --- | --- | --- | --- | --- | --- | --- | --- |
| **SOT 01** | 62 | M | Heart | 22 | CNI | High | **-** |
| **SOT 03** | 77 | F | Kidney | 10 | CNI | High | **-** |
| **SOT 04** | 78 | M | Heart+Kidney^1^ | 23 | CNI+mTORI | High | SCC^4^  BCC^5,6^ |
| **SOT 05** | 65 | F | Heart | 16 | CNI+mTORI | High | **-** |
| **SOT 06** | 60 | M | Kidney | 28 | CNI | High | Marginal-zone NHL^7^ |
| **SOT 07** | 65 | M | Kidney | 5 | CNI^2^ | High | BCC^5,6^ |
| **SOT 08** | 57 | M | Kidney | 28 | CNI | High | BCC^5,6^ |
| **SOT 11** | 61 | M | Kidney | 4 | CNI^2^ | High | Renal Carcinoma^8^ |
| **SOT 14** | 64 | M | Kidney | 6 | CNI+mTORI | High | SCC^4^ |
| **SOT 16** | 41 | F | Kidney | 4 | CNI^2^ | Low | **-** |
| **SOT 17** | 64 | M | Liver | 8 | mTORI | High | **-** |
| **SOT 18** | 51 | M | Kidney | 17 | CNI | High | **-** |
| **SOT 19** | 50 | M | Kidney | 6 | CNI | High^3^ | **-** |
| **SOT 31** | 63 | F | Kidney | 25 | CNI | High | **-** |
| **SOT 32** | 44 | M | Kidney+Kidney^1^ | 25 | CNI | High | BCC^5,6^ |
| **SOT 34** | 67 | F | Kidney | 21 | CNI | High | Melanoma in situ^4^ |
| **SOT 35** | 54 | F | Kidney | 15 | CNI | High | BCC^5,6^ |
| **SOT 36** | 54 | M | Heart | 11 | CNI+mTORI | High | **-** |
| **SOT 37** | 70 | M | Kidney | 7 | CNI | High | SCC^4^ |
| **SOT 40** | 59 | F | Kidney | 3 | CNI+mTORI | High | BCC^5^ |
| **SOT 41** | 50 | F | Kidney | 11 | CNI | High | **-** |
| **SOT 42** | 47 | F | Kidney | 4 | CNI | Low | **-** |
| **SOT 43** | 59 | F | Kidney | 12 | mTORI | High | BCC^5,6^ |
| **SOT 44** | 52 | F | Kidney | 5 | CNI+mTORI | Low | **-** |
| **SOT45** | 55 | M | Kidney | 9 | CNI | High^3^ | **-** |
| **SOT 46** | 59 | M | Heart | 10 | CNI | High | **-** |
| **SOT 49** | 67 | M | Heart | 14 | CNI | High | BCC^5,6^ |
| **SOT 50** | 69 | M | Kidney | 11 | CNI | High | **-** |
| **SOT 53** | 52 | M | Kidney | 15 | CNI | High | **-** |
| **SOT 56** | 78 | M | Heart | 16 | mTORI | High | **-** |
| **SOT 57** | 58 | M | Kidney | 14 | CNI | High | **-** |
| **SOT 59** | 58 | M | Kidney | 8 | CNI | High^3^ | **-** |
| **SOT 62** | 64 | M | Liver | 13 | mTORI | High | **-** |
| **SOT 65** | 56 | M | Kidney | 15 | CNI | High | **-** |
| **SOT 67** | 49 | F | Kidney | 8 | CNI+mTOR | High^3^ | **-** |
| **SOT 72** | 32 | F | Kidney | 15 | CNI | High | **-** |
| **SOT 73** | 69 | F | Kidney | 4 | CNI | High | **-** |
| **SOT 74** | 35 | M | Kidney | 5 | CNI | Low | **-** |
| **SOT 80** | 80 | M | Kidney | 12 | CNI | High | BCC^5,6^ |
| **SOT 82** | 31 | M | Kidney | 1 | CNI | Low | **-** |
| **SOT 83** | 58 | M | Kidney | 9 | CNI | High | SCC^5,6^ |
| **SOT 86** | 72 | M | Kidney | 15 | CNI^2^ | High | **-** |
| **SOT 93** | 71 | M | Kidney | 19 | CNI | High | **-** |
| **SOT 95** | 72 | F | Kidney | 5 | CNI^2^ | High | **-** |
| **SOT 97** | 68 | F | Kidney | 5 | CNI | High | **-** |
| **SOT 98** | 78 | M | Kidney | 6 | CNI | High | SCC^4,5,6^ |
| **SOT 102** | 53 | M | Kidney | 6 | CNI | Low | **-** |
| **SOT 104** | 50 | M | Kidney+Kidney^1^ | 15 | CNI^2^ | High | **-** |
| **SOT 109** | 68 | F | Kidney | 5 | CNI | High | **-** |

IS, immune suppression; M, male; F, female; CNI, calcineurin inhibitors; mTORI, mammalian target of rapamycin inhibitors; BCC, Basocellular carcinoma; NHL, Non-Hodgkin Lymphoma; SCC, Squamous cell carcinoma; ^1^, metachronous transplant; ^2^, adjunctive pre-transplant immunosuppressive therapy; ^3^, smoking abuse; ^4^, pTis; ^5^, pT1N0M0; ^6^, multifocal tumor; ^7^, IIE (non gastric extranodal disease) A; ^8^, Stage IV.

Supplementary Table 2: Characteristics of the clinical and biological parameters clustered according to the area under the ROC curve

|  | **AUROC (95% C.I.)** | **p-value** | **Optimal cut-off** | **Sensibility (95% C.I.)** | **Specificity (95% C.I.)** |
| --- | --- | --- | --- | --- | --- |
| **Time of Immunosuppression** | 0.57 (0.42-0.71) | 0.46 | ˃18.83 years | 31.3 (11.0-58.7) | 93.9 (79.8-99.3) |
| **EBV-DNA, copies/mL** | 0.58 (0.41-0.75) | 0.35 | >29 copies/mL | 62.5 (35.4-84.8) | 57.6 (39.2-74.5) |
| **T cell responses against EBV** | 0.56 (0.41-0.70) | 0.54 | ≤106 sfu/10^5 PBMCs | 64.3 (35.1-87.2) | 60.6 (42.1-77.1) |
| **T cell responses against CMV** | 0.56 (0.41-0.70) | 0.50 | ≤1097 sfu/10^5 PBMCs | 100.0 (76.8-100.0) | 21.21 (9.0-38.9) |
| ***TERT* mRNA** | 0.70 (0.60-0.82) | 0.03 | >97.73 copies/mL | 53.3 (26.6-78.7) | 93.9 (79.8-99.3) |
| **T cell responses against TERT** | 0.56 (0.41-0.70) | 0.53 | >8 sfu/10^5 PBMCs | 71.4 (41.9-91.6) | 48.5 (30.8-66.5) |
| **T cell responses against SURVIVIN** | 0.53 (0.38-0.68) | 0.73 | >13 sfu/10^5 PBMCs | 50.0 (23.0-77.0) | 63.6 (45.1-79.6) |

AUROC, area under the ROC curve; sfu/10^5 PBMCs, spot forming units/10^5 Peripheral Blood Cells.
